# First Thai Case of Lethal Desbuquois Dysplasia Type I Caused by Novel Compound Heterozygous *CANT1* Mutations: Expanding the Molecular Spectrum

**DOI:** 10.1155/crig/9550632

**Published:** 2026-02-09

**Authors:** Supitcha Thamissarakul, Teeraphorn Boonswang, Sethapong Lertsakulbunlue, Siriluk Khumsui, Boonchai Boonyawat

**Affiliations:** ^1^ Department of Pediatrics, Chonburi Hospital, Chonburi, Thailand; ^2^ Department of Radiology, Chonburi Hospital, Chonburi, Thailand; ^3^ Department of Pharmacology, Phramongkutklao College of Medicine, Bangkok, Thailand, pcm.ac.th; ^4^ Department of Pediatrics, Phramongkutklao College of Medicine, Bangkok, Thailand, pcm.ac.th

## Abstract

Desbuquois dysplasia Type 1 (DBQD1) is an extremely rare autosomal recessive skeletal dysplasia characterized by severe short stature, joint laxity, distinct facial dysmorphism, and advanced carpotarsal ossification. Here, we report the first Thai patient diagnosed with classical lethal DBQD1. A 38‐week male infant presented with multiple dysmorphic features, micromelia, joint dislocations, narrow thorax, and respiratory insufficiency leading to death at seven months of age. Radiographic findings revealed hallmark features, including a “Swedish key” appearance of the proximal femur and characteristic hand and foot anomalies. Whole exome sequencing identified compound heterozygous missense variants of c.505G > *A* (p.Asp169Asn) and c.1028G > *T* (p.Gly343Val) in the *CANT1* gene. The 3D structural modeling revealed that both variants reside in conserved regions, with predicted effects on calcium binding and protein folding, resulting in impaired enzymatic function and proteoglycan synthesis. Genetic counseling was provided to the family, and prenatal or preimplantation genetic diagnosis was discussed as an option for future pregnancies. Our report expands the mutational spectrum of the *CANT1* gene, contributing to a better understanding of DBQD1’s clinical and molecular presentation, particularly in Southeast Asian populations.

## 1. Introduction

Desbuquois dysplasia (DBQD) is an extremely rare autosomal recessive osteochondrodysplasia with an incidence of approximately 1 in 1,000,000 and is classified within the group of multiple joint dislocations according to the nosology of genetic skeletal disorders [1]. DBQD was first described in 1966 by Desbuquois et al. [2]. The clinical manifestations of DBQD include pre‐ and postnatal growth retardation, micromelic dwarfism, facial dysmorphism, narrowing chest wall, vertebral and metaphyseal abnormalities, generalized joint laxity, and advanced carpotarsal ossification [1–3]. The radiographic hallmark of DBQD is a “monkey wrench” or “Swedish key” appearance of the proximal femur characterized by a flat proximal femoral metaphysis with a medial spike and an exaggerated lesser trochanter. DBQD has been classified into two types based on the presence (DBQD1, MIM 251450) and absence (DBQD2, MIM 615777) of characteristic hand abnormalities [3].

DBQD1 is caused by homozygous or compound heterozygous mutations in the calcium‐activated nucleotidase 1 (*CANT1*) gene located at 17q25.3 [1]. *CANT1* is expressed in chondrocytes and encodes an extracellular CANT1 protein that acts as a calcium‐dependent tri‐ and diphosphate nucleotidase. DBQD2 is primarily caused by mutations in the xylosyltransferase 1 (*XYLT1*) gene located on Chromosome 16p12 and is now recognized as Baratela–Scott syndrome (*XYLT1*‐associated DBQD) [1]. *XYLT1* plays a key role in proteoglycan (PG) synthesis. However, *CANT1* mutations were also responsible for some DBQD2 individuals [4]. To date, only 54 cases of *CANT1*‐related DBQD1 have been reported in the literature [3, 4]. Nevertheless, no DBQD1 individual has been described in Southeast Asian populations.

Herein, we report the first Thai patient with DBQD1 and the classical lethal phenotype, along with pathognomonic radiology, and identified compound heterozygous of two novel missense variants in the *CANT1* gene by whole exome sequencing (WES). Sanger sequencing also verified the carrier status in both parents. Together, these findings expand the allelic heterogeneity and ethnic spectrum of *CANT1*‐related chondrodysplasia.

## 2. Case Report

A 38‐week male infant was the first child of healthy nonconsanguineous parents. Both parents were 30 years old at the time of delivery. Prenatal ultrasound at 33 weeks of gestation revealed significantly short long bones (< 5^th^ percentile for gestational age) and a small thorax. The fetus was diagnosed as having lethal skeletal dysplasia. He was delivered by cesarean section due to breech presentation at the Chonburi Province hospital. Pregnancy was also complicated by gestational diabetes. The family history was unremarkable.

**FIGURE 1:**
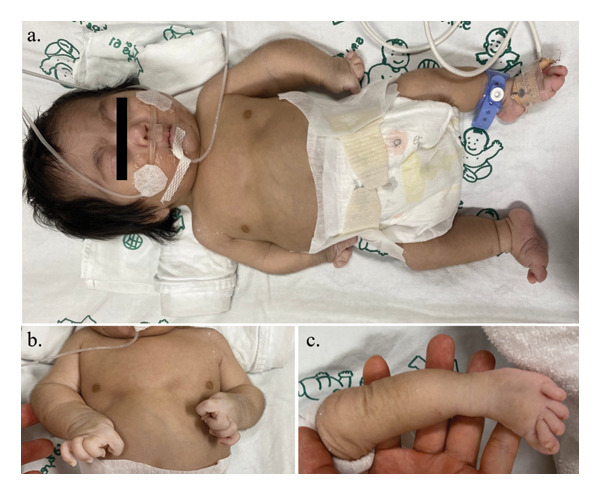
Clinical characteristics of the DBQD1 patient in this study. (a) Full‐body view shows a relatively round face, midface hypoplasia, short sternum, short bowed limbs, and rocker bottom feet. (b) Ulnar clinodactyly of the 3rd–5th digits, radial clinodactyly of the index finger and low‐set “hitchhiker” thumbs and (c) overriding of the 2nd toe on both feet.

Physical examination after birth revealed a weight of 2232 g (Z‐score −2.16), length 38 cm (Z‐score −4.80), head circumference 29 cm (Z‐score −3.38), and an upper/lower body ratio of 2.17. Dysmorphic facial features, including a flat round face, prominent eyes, depressed nasal bridge with maxillary hypoplasia, micrognathia, and a short neck, were detected (Figure 1). Skeletal examination showed a narrow chest with a short sternum, short bowed limbs, clasped thumbs, brachydactyly and clinodactyly of all fingers, short flat feet with an overriding second toe, and multiple joint dislocations. He was intubated after birth due to respiratory insufficiency from thoracic cage dysplasia and put on mechanical ventilation until he died at 7 months of age. Chromosome analysis revealed 46,XY. He was initially diagnosed with acromelic skeletal dysplasia.

**FIGURE 2:**
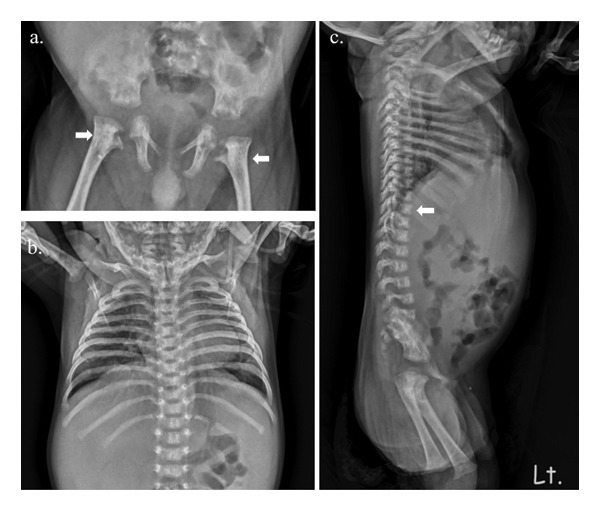
Skeletal radiography including pelvis (AP) and spine (AP and lateral) of the patient. (a) Hip and proximal femur shows “monkey wrench” characteristic (arrow). (b) Note narrow upper thorax with wavy ribs. (c) Coronal clefts of lumbar spines (arrow).

**FIGURE 3:**
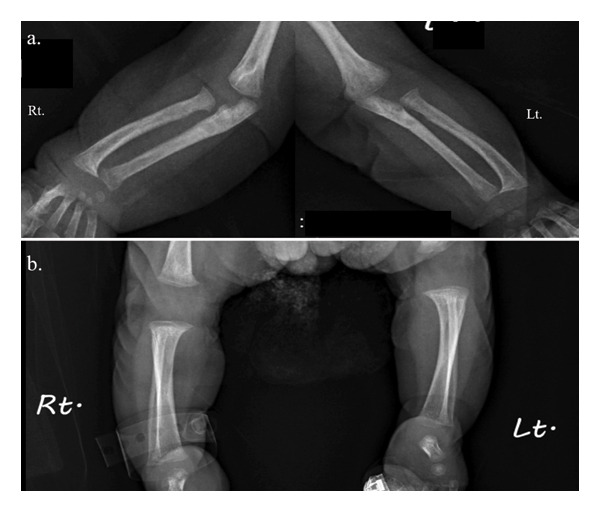
Skeletal radiography of the patient’s long bone (AP). (a) Forearms showed widening of the metaphysis and mild bowing of bilateral radius and ulna. (b) Legs showed widening of the metaphysis and mild bowing of bilateral tibia, overgrowth of bilateral proximal fibula.

The skeletal survey revealed flat metaphyses with prominent lesser trochanters of both proximal femurs, producing a characteristic “Swedish key” appearance and flat bilateral acetabular roofs. Coronal clefts in the spine were seen. Narrowing of the upper thorax with wavy ribs was found (Figure 2). Forearms and legs showed widening of the metaphyses and mild bowing of long bones (Figure 3). The cranium was relatively large.

## 3. Materials and Methods

### 3.1. Exome Sequencing

This study was approved by the Institutional Review Board of the Chonburi Hospital. After informed consent was obtained, genomic DNA of the patient and both parents were extracted from peripheral blood samples according to the manufacturer’s protocol. WES was performed on the NovaSeq 6000 platform (Illumina, San Diego, CA, USA) using the SureSelect Human All Exon V7 (Agilent Technologies, Santa Clara, CA, USA). DNA reads were mapped against the human genome reference (hg19/GRCh37). The total number of reads and coverage of our WES sample were 45 million reads and 97.2% of the targeted regions at 10x coverage. The reference sequences were NM_001159773.2 for *CANT1* cDNA and NP_001153244.1 for the CANT1 amino acid position. The pathogenicity of rare variants was classified following the ACMG 2015 guidelines [5]. Sanger sequencing was performed to verify any identified variants. Multiple in silico models such as Polyphen, SIFT, MutationTaster, and CADD were used for predicting the identified missense variants.

### 3.2. 3D Protein Structure Modeling of CANT1

The 3D structure of the CANT1 protein was utilized for analyzing the potential effects of the identified missense variants [6]. The sequence of the CANT1 protein was extracted from the Protein Data Bank (PDB: 2h2u) and UniProtKB (Q8WVQ1). The online website ProtVar (https://www.ebi.ac.uk/ProtVar) was used for predicting the 3D structure modeling of the CANT1 protein [6].

## 4. Results

**FIGURE 4:**
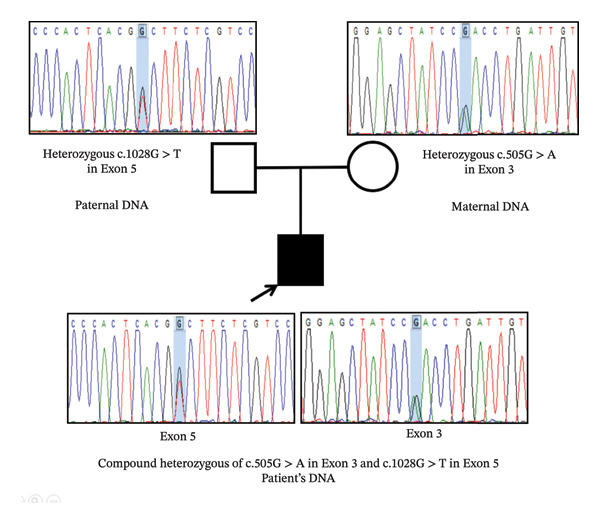
Sanger sequencing of Exon 3 and Exon 5 of the *CANT1* gene reveals compound heterozygous of c.505G > *A* variant in Exon 3 and c.1028G > *T* variant in Exon 5 in the patient’s DNA, which were inherited from both heterozygous parents.

A compound heterozygous of two missense variants, c.505G > *A* (p.Asp169Asn) in Exon 3 and c.1028G > *T* (p.Gly343Val) in Exon 5, of the *CANT1* gene was identified in the patient’s DNA. The former c.505G > *A* variant was inherited from the mother; whereas, the latter c.1028G > *T* variant was inherited from the father (Figure 4). According to the ACMG classification guidelines [5], both variants were classified as “variant of uncertain significance” according to the PM2, PP2, PP3, and PP4 criteria. However, the presence of these two variantsin *trans* also provides supportive evidence of pathogenicity, consistent with the autosomal recessive inheritance pattern of DBQD1.

**FIGURE 5:**
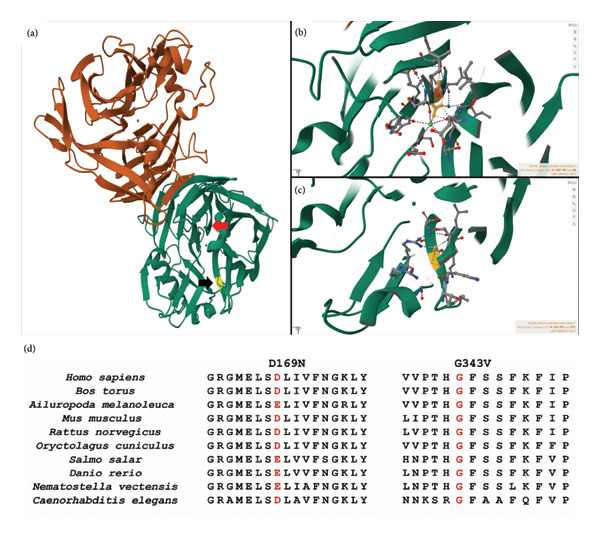
Predicted 3D structure of the CANT1 protein. (a) The 3D structure of the CANT1 protein (PDB: 2h2u) was demonstrated. The location of aspartic acid (red arrow) at Position 169 (D169) and glycine (black arrow) at Position 343 (G343) was illustrated. (b) The D169 (shown in yellow) is one of the calcium‐binding residues of the CANT1 protein that directly interacts with the calcium ion (shown as a small green ball). (c) The G343 (shown in yellow) is located in the beta‐strand of the CANT1 protein. (d) The D169N and G343V missense variants are located in the very highly conserved region of the CANT1 protein.

The 3D structure of the CANT1 protein is shown in Figures 5(a), 5(b), 5(c), and 5(d) [6]. Both missense variants are located in evolutionarily conserved regions of the CANT1 protein (Figure 5(d)). The c.505G > *A* variant is located on the calcium‐binding site residue, which is highly conserved to only aspartic or glutamic acid (Figures 5(b) and 5(d)). Changing from aspartic acid, which normally has an acidic carboxylate (‐COO‐) side chain, to asparagine, whose side chain is neutral carboxamide (‐NH2), is expected to have a disruptive effect on calcium binding, with a 96% reduced enzymatic activity as predicted by an in silico model. The c.1028G > *T* variant is located in the beta‐strand and is a very highly conserved glycine residue (Figures 5(b) and 5(d)). The replacement of glycine, which is polar and hydrophilic, by nonpolar and hydrophobic valine is expected to be probably deleterious. The CANT1 protein was predicted to be destabilized by both variants, indicating the importance of both loci for the protein function.

## 5. Discussion

DBQD is a rare autosomal recessive chondrodysplasia with variable severities, ranging from the most severe form, which is usually lethal in infancy, to the milder form, which sometimes survives into adulthood [3, 4]. In this study, we reported the first Thai DBQD patient presented with the classical features of the severe lethal form of DBQD, including prenatal short stature, micromelia, facial dysmorphism, multiple joint laxity, and thoracic dysplasia resulting in respiratory insufficiency, which was the major cause of early death in our patient. Additionally, a skeletal survey serves a crucial role in differentiating disorders associated with lethal skeletal dysplasia. DBQD typically exhibits the characteristic radiographic appearance known as the “Swedish key” appearance of the proximal femur, characterized by a flat proximal femoral metaphysis with a medial spike and an exaggerated lesser trochanter [3, 4]. This typical skeletal feature was also demonstrated in our patient.

In this study, a compound heterozygous of two missense variants, c.505G > *A* (p.Asp169Asn) in Exon 3 and c.1028G > *T* (p.Gly343Val) in Exon 5, of the *CANT1* gene was identified in our DBQD1 patient. Both variants were initially classified as “variant of uncertain significance” by ACMG classification according to several reasons [5]. First, both variants were either absent or rarely present in the public SNP databases (PM2). The Allele A of c.505G > *A* was described in dbSNP (rs780464924) with an allele frequency of 0.000004 in gnomAD and was identified only in the heterozygous state. In contrast, the c.1028G > *T* allele has never been identified in any population databases. Second, both missense variants were predicted to be deleterious by multiple in silico analyses and 3D structure modeling of the protein (PP3). The substitution of aspartic acid by asparagine at amino acid 169, which is one of the calcium‐binding residues, directly affects the enzymatic activity of the CANT1 protein. The replacement of glycine by valine at amino acid 343 was predicted to be deleterious due to the very high conservation of this position and the biophysical differences between wild‐type and mutant amino acids [6]. Third, various types of *CANT1* mutations, including missense, nonsense, frameshift, and splice‐site mutations, have been reported in previous literature. Missense mutations account for over 35% of all mutations [3, 4], and missense mutations in the *CANT1* gene have a lower rate of being benign (PP2). Finally, DBQD1 is a genetic disorder characterized by a highly specific phenotype and is caused by mutations in the *CANT1* gene alone (PP4) [3, 4]. In addition, Sanger sequencing confirmed that both variants were inherited from both heterozygous parents, compatible with the autosomal recessive inheritance of DBQD1.

CANT1 is a protein functioning as a calcium‐activated tri‐ and diphosphate nucleotidase, with its primary role in hydrolyzing mainly uridine diphosphate (UDP), followed by guanosine diphosphate (GDP), uridine triphosphate (UTP), and guanosine triphosphate (GTP). Hydrolyzing UDP plays a crucial role in maintaining the nucleotide balance necessary for the glycosylation process, including PG synthesis within the endoplasmic reticulum (ER) and Golgi apparatus [7, 8]. Mutations in the *CANT1* gene result in the dysfunction of the CANT1 protein, leading to UDP accumulation and disruption of PG synthesis, which is crucial for extracellular matrix (ECM) integrity, particularly in cartilage [7, 8]. This biosynthetic defect causes deregulated chondrocyte proliferation and maturation in the growth plate, resulting in reduced skeletal growth, which is the main pathogenesis of DBQD [8]. Prior functional studies have shown that *CANT1* missense variants reduce UDP–nucleotidase activity and impair PG/glycosaminoglycan synthesis in patient fibroblasts and recombinant systems, with *CANT1* animal models recapitulating disorganized growth plates and skeletal dysplasia [8, 9]. In this context, our p.Asp169Asn, which affects a calcium‐binding residue essential for catalysis, and p.Gly343Val—at a highly conserved position—map to regions previously implicated in the loss of enzymatic function, providing mechanistic concordance with the established pathogenic alleles. This comparison supports our assertion that these changes broaden the allelic and ethnic spectrum of *CANT1*‐related DBQD1, while acknowledging that further formal functional validation would refine the result.

Considering our findings, DBQD1 requires a multidisciplinary plan centered on symptom and complication management, particularly for respiratory failure, the leading cause of death. Given its high mortality rate, ranging from 28% to 33%, and the high likelihood of death within the first year in severe cases, early diagnosis and appropriate management are essential [3, 10]. Therefore, recognizing lethal DBQD1 early by its pathognomonic skeletal signs (notably the “Swedish key” femur) and confirmation of a definitive *CANT1* gene diagnosis enables actionable counseling (25% autosomal‐recessive recurrence risk) and prompt timely discussion of prenatal or preimplantation genetic testing for future pregnancies.

## 6. Conclusion

In this study, we reported the first Thai patient with classical severe DBQD1. A compound heterozygous of two novel missense mutations in the *CANT1* gene was identified in the patient and was inherited from both heterozygous parents. This finding highlights the genetic heterogeneity of *CANT1* mutations that cause DBQD1. Although DBQD1 remains incurable, a correct genetic diagnosis potentially mitigates the disease impact for the family, especially in the prevention of future pregnancies.

## Author Contributions

Supitcha Thamissarakul, Teeraphorn Boonswang, Sethapong Lertsakulbunlue, Siriluk Khumsui, and Boonchai Boonyawat contributed to drafting and writing the manuscript. Supitcha Thamissarakul and Boonchai Boonyawat are involved in the patient’s clinical care. Sethapong Lertsakulbunlue, Siriluk Khumsui and Boonchai Boonyawat performed the molecular and data analysis. All authors contributed to the manuscript.

## Funding

The authors reported no funding associated with the work featured in this article.

## Disclosure

All authors approved the final version of the manuscript.

## Ethics Statement

This case report was approved by the Medical Department Ethics Review Committee for Research in Human Subjects, Institutional Review Board, Chonburi Hospital Ethics Committee (code 8/67/s/h3), following international guidelines such as the Declaration of Helsinki, the Belmont Report, CIOMS Guidelines, and the International Conference on Harmonization of Technical Requirements for Registration of Pharmaceuticals for Human Use–Good Clinical Practice. Informed consent for participation and publication was obtained from the parents, with permission from the Institutional Review Board and the Chonburi Hospital Ethics Committee.

## Conflicts of Interest

The authors declare no conflicts of interest.

## Data Availability

The data from this study can be made available upon a reasonable request to the corresponding author.
